# Association of male pattern baldness with angiographic coronary artery disease severity and collateral development

**DOI:** 10.1007/s12471-015-0688-3

**Published:** 2015-04-01

**Authors:** I. Sari, K. Aykent, V. Davutoglu, M. Yuce, O. Ozer, M. Kaplan, H. Alici, S. Ercan, M. Sunbul, K. Tigen

**Affiliations:** 1 Department of Cardiology, School of Medicine, Marmara University, Istanbul, Turkey; 2 Department of Cardiology, School of Medicine, Gaziantep University, Gaziantep, Turkey; 3 Marmara University Education and Research Hospital, Pendik, Istanbul, Turkey

**Keywords:** Baldness, Alopecia, Coronary artery disease, Atherosclerosis, Gensini, Rentrop

## Abstract

**Objective:**

We aimed to investigate whether there is an association between male pattern baldness and angiographic coronary artery disease (CAD) severity and collateral development, which has not been reported previously.

**Methods:**

Coronary arteriograms, CAD risk factors, lipid parameters and presence and severity of baldness in 511 male patients were prospectively evaluated. Baldness was classified into five groups. Severity of CAD was evaluated with the Gensini scoring system and collateral development with Rentrop scores.

**Results:**

Although subjects with a higher Gensini score had more frequent and severe baldness, they were older than the group with lower Gensini scores. Bald patients had a higher Gensini score when compared with their non-bald counterparts. In univariate analysis, age more than 60, body mass index more than 30, smoking and baldness were predictors of high Gensini scores. In multivariate analysis, only age more than 60, body mass index more than 30 and smoking were independent predictors of a high Gensini score. There were no differences in terms of presence and severity of baldness in subjects with and without adequate collateral development.

**Conclusions:**

There was no relation between presence, severity and age of occurrence of male pattern baldness and Gensini and Rentrop scores, which are important measures of presence and severity of CAD.

## Introduction

Coronary artery disease (CAD) is the leading cause of morbidity and mortality throughout the world [1, 2]. Classical CAD risk factors are not enough to explain all coronary events. Therefore, researchers are searching for potential novel risk factors for CAD.

Male pattern baldness, also called androgenetic alopecia, is responsible for the majority of hair loss in male individuals [3–5]. It generally starts at the second or third decade and is characterised by varying degrees of thinning/hair loss primarily at the frontal area and vertex of the scalp.

Several epidemiological studies have shown an association between androgenetic alopecia and CAD [6–10]. Among these, the Framingham Heart Study revealed an association between progression of hair loss during adulthood and CAD in male individuals, although there was no relationship with the extent of baldness [7]. In a case-control study, Lesko et al. [9] reported that vertex baldness was associated with threefold higher risk of myocardial infarction among men younger than 55 years. Several other studies reported the presence of an association between androgenetic alopecia and CAD [11–13]. However, results of several other studies argue against this relationship [14, 15].

Clarifying the potential relationship between androgenetic alopecia and CAD might help to better understand the pathophysiology of coronary heart disease. Although several epidemiological studies have shown an association between male pattern baldness and atherosclerosis, it has never been studied by investigating angiographic presence and severity of CAD. We therefore aimed to shed light on this issue by investigating whether there is an association between male pattern baldness and angiographic CAD severity and collateral development.

## Material and methods

### Study population and protocol

The study complies with the principles outlined in the Declaration of Helsinki. The study was approved by the local ethics committee, and all participants gave written informed consent before participating. Patients scheduled for an elective coronary arteriogram in the Cardiology Department of Gaziantep University Hospital were invited to participate.

Demographic data on the study population including age, sex, height, weight, history of diabetes mellitus, hypertension, smoking status, family history of CAD, lipid parameters and creatinine levels were recorded. Before coronary arteriogram, the presence and severity of hair loss was recorded according to the criteria mentioned later in the text. Severity of CAD was determined according to the Gensini score and collateral flow was graded according to the Rentrop score. Exclusion criteria were female sex, history of coronary artery bypass surgery, history of acute coronary syndrome in the past 4 weeks, other types of alopecia and patients with known hormonal problems.

### Coronary arteriogram

Coronary arteriogram was performed via the femoral, radial or brachial artery using Judkins technique (Integris H 5000, Philips Medical Systems, the Netherlands). Coronary artery stenosis severity (Gensini and Rentrop scores) was estimated visually by two independent observers who were blinded to clinical information on the patients. Then the mean of the two measures was calculated.

### Evaluation of coronary artery disease severity

Coronary arteries were considered to be normal (0: no stenosis), obstructed (25, 50, 75, 90 or 99 % stenosis) or 100 % occluded, according to the maximum obstruction that was observed in any projection. The severity of coronary atherosclerosis was classified according to the Gensini score, which grades narrowing of the lumen as 1 for 1–25 % stenosis, 2 for 26–50 %, 4 for 51–75 %, 8 for 76–90 %, 16 for 91–99 % and 32 for total occlusion [16]. This score was multiplied by a factor that accounted for the importance of a lesion’s position in the coronary arterial tree: 5 for the left main coronary artery; 2.5 for the proximal left anterior descending coronary artery or proximal circumflex artery; 1.5 for the mid left anterior descending coronary artery; 1 for the proximal right coronary artery, distal left anterior descending coronary artery, obtuse marginal artery or posterior lateral artery; and 0.5 for other stenoses. The severity of disease was expressed as the sum of the scores for the individual lesions. By definition, a Gensini score of 20 or more was considered to be severe CAD.

### Evaluation of collateral development

Collateral vessels were graded according to the Rentrop classification: 0: no filling of any collateral vessels, 1: filling of side branches of the artery to be perfused by collateral vessels without visualisation of the epicardial segment; 2: partial filling of the epicardial artery by collateral vessels; and 3: complete filling of the epicardial artery by collateral vessels [17]. By definition, a Rentrop score of 2 or more was accepted as well-developed collaterals.

### Classification of hair loss (baldness scale)

We used the modified Hamilton grading system, as it was used by the majority of the studies: Grade 1: no hair loss; Grade 2: frontal baldness only; Grade 3: mild vertex baldness; Grade 4: moderate vertex baldness and Grade 5: severe vertex baldness as previously described [12, 18]. Each investigator had a chart illustrating these five different hair types, as depicted by Lotufo et al. [12] and decided to which grade the individual patient’s hair status fits best. Additionally, investigators asked each participant which category best describes his hair status when he was 35 years old.

### Statistical analysis

According to the power calculation, to provide 95 % power at 5 % significance to detect a 4 % mean absolute difference in baldness and Gensini score, 306 subjects were necessary. To provide 80 % power at 5 % significance to detect a 4 % mean absolute difference in baldness and Rentrop score, 149 subjects were necessary. Continuous data were expressed as mean ± standard deviation, while categorical data were presented as number or percentage of patients. Chi-square test was used for comparison of categorical variables, while the Mann–Whitney *U* test was used to compare continuous variables. Correlation analyses were performed with Pearson’s test. Multivariate logistic regression analysis (backward) was performed to determine the independent predictors of a high Gensini score. A value of *p* < 0.05 was considered statistically significant. All analyses were carried out with SPSS version 15.0 software for Windows (SPSS Inc., Chicago, Illinois, USA).

## Results

A total of 511 male patients (mean age: 58.6; range: 33–84) were included in the study. Gensini score was available in 511 patients, whereas the Rentrop score was available in 136 patients. Table 1 presents the characteristics of the study population.

**Table 1:** Characteristics of the study population

	*N*	
Age	511	58.6 ± 12.0
Hypertension (%)	511	182 (35.7)
Diabetes (%)	511	106 (20.9)
Smoking (%)	511	330 (64.6)
Family history (%)	511	190 (37.2)
Baldness scale	511	2.84 ± 1.54
Gensini score	511	41.3 ± 40.3
Rentrop score	136	1.84 ± 1.09
Body mass index (kg/m^2)^	509	27.1 ± 4.4
Creatinine (mg/dl)	478	1.02 ± 0.94
Low-density lipoprotein (mg/dl)	419	111.2 ± 33.5
High-density lipoprotein (mg/dl)	419	35.5 ± 15.9
Total cholesterol (mg/dl)	419	176.0 ± 42.0
Triglyceride (mg/dl)	419	173.3 ± 128.0

Data are expressed as mean ± standard deviation or percentages

Comparison of demographic, clinical and laboratory characteristics of the subjects according to Gensini score is expressed in Table 2. Those with a higher Gensini score (≥ 20) were older, had a higher level of triglycerides and androgenetic alopecia was more common among them. The hair status at the age of 35 was similar between the groups with high and low Gensini scores. Additionally, the rest of the laboratory results and demographic characteristics were similar between the groups with high and low Gensini scores. When we compared bald (modified Hamilton score ≥ 2) and non-bald (modified Hamilton score = 1) subjects, bald patients had a higher Gensini score (44.2 ± 42.1 vs. 33.0 ± 35.3; *p* = 0.003). Smoking rate was higher in the group with higher Gensini scores (*p* = 0.002). There were no differences in terms of presence of diabetes, hypertension and family history of CAD between the groups with high and low Gensini scores.

**Table 2:** Comparison of demographic, clinical and laboratory characteristics of the subjects according to Gensini score

Parameter	*N*	Gensini score < 20	*N*	Gensini score ≥ 20	*p* Value
Age	196	56.3 ± 12.2	315	60.1 ± 11.7	**0**.**001**
Baldness scale	196	2.63 ± 1.5	314	2.97 ± 1.5	**0**.**016**
Baldness scale at age 35	170	1.58 ± 0.9	293	1.52 ± 0.9	0.4
Creatinine, mg/dl	179	1.03 ± 1.17	295	1.01 ± 0.78	0.8
Triglycerides, mg/dl	161	154.5 ± 81	263	185.5 ± 149	**0**.**006**
HDL, mg/dl	152	36.7 ± 9.9	263	34.7 ± 18.5	0.1
LDL, mg/dl	158	112 ± 30.8	264	110.7 ± 35.1	0.7
Total cholesterol, mg/dl	158	175.8 ± 37.6	264	176.2 ± 44.7	0.6
BMI, kg/m^2^	195	27 ± 4.2	310	27.4 ± 4.3	0.2

Data are expressed as mean ± standard deviation or as number of patients

*HDL* high-density lipoprotein, *LDL* low-density lipoprotein, *BMI* body mass index

Comparison of demographic, clinical and laboratory characteristics of the subjects according to the Rentrop score is expressed in Table 3. There were no differences in terms of presence and severity of baldness, hair status at the age of 35, demographic characteristics and laboratory findings in subjects with (Rentrop score ≥ 2) and without (Rentrop score 0 or 1) adequate collateral development. Additionally, the rate of smoking, presence of diabetes, hypertension and family history of CAD were similar in subjects with and without adequate collateral development.

**Table 3:** Comparison of demographic, clinical and laboratory characteristics of the subjects according to Rentrop score

Parameter	*N*	Rentrop < 2	*N*	Rentrop ≥ 2	*p* Value
Age	42	58.2 ± 12.6	94	60.6 ± 12.1	0.3
Baldness scale	42	3 ± 1.4	94	2.9 ± 1.4	0.7
Baldness scale at age 35	42	1.67 ± 1	94	1.55 ± 1	0.5
Creatinine, mg/dl	42	0.98 ± 0.38	85	0.97 ± 0.28	0.8
Triglycerides, mg/dl	35	195.5 ± 142.4	77	194.5 ± 158.3	0.9
HDL, mg/dl	35	41.5 ± 45.5	77	32.5 ± 9.2	0.2
LDL, mg/dl	35	111.1 ± 33.9	78	110.7 ± 41.6	0.9
Total cholesterol, mg/dl	35	176.3 ± 31.9	77	174.2 ± 55.2	0.8
BMI, kg/m^2^	42	26.9 ± 4.2	90	27.4 ± 3.7	0.5

Data are expressed as mean ± standard deviation or as number of patients

*HDL* high-density lipoprotein, *LDL* low-density lipoprotein, *BMI* body mass index

Of 511 patients, 59 had normal coronary arteries (zero-vessel disease), 171 had one-vessel disease, 176 had two-vessel disease and 105 had triple-vessel disease. The mean number of diseased vessels was higher in bald subjects when compared with their non-bald counterparts (1.71 ± 0.9 vs. 1.44 ± 0.9; *p* = 0.004). The number of diseased vessels correlated with the severity of baldness (*r* = 0.125; *p* = 0.005). The mean number of diseased vessels was lower in the group with adequate collateral development when compared with those without adequate collateral development (2.47 ± 0.5 vs. 2.69 ± 0.4; *p* = 0.028). The number of diseased vessels was inversely correlated with Rentrop score (*r* = − 0.226; *p* = 0.008).

In univariate analysis, age more than 60, body mass index more than 30, smoking and baldness were among the predictors of a high Gensini score. In multivariate analysis, only age more than 60 (*p* < 0.001; odds ratio 2.590; 95 % confidence interval 1.637–4.098), smoking (*p* < 0.001; odds ratio 2.385; 95 % confidence interval 1.496–3.802) and body mass index more than 30 (*p* = 0.012; odds ratio 1.937; 95 % confidence interval:1.154–3.252) were independent predictors of a high Gensini score (Table 4). After adjusting for age, presence and severity of baldness was not an independent predictor of a high Gensini score.

**Table 4:** Independent predictors of Gensini score > 20 (multivariate logistic regression analysis)

Variable	Odds ratio	95 % Confidence interval
Age > 60	2.590	1.637–4.098
Smoking	2.385	1.496–3.802
BMI > 30 kg/m^2^	1.937	1.154–3.252

*BMI* body mass index

## Discussion

In the present study, we aimed to investigate whether there is an association between male pattern baldness and angiographic CAD severity and collateral development, which has not been reported previously. According to our findings, although subjects with higher Gensini scores had more frequent and severe baldness, they were older than the group with lower Gensini scores. After adjusting for age, presence and severity of baldness lost its value in predicting the severity of CAD. Additionally, there were no differences in terms of presence and severity of baldness in subjects with and without adequate collateral development.

Because CAD is the leading cause of morbidity and mortality worldwide, researchers aim to investigate potential novel risk factors or associated conditions which might enable the early detection of CAD. Male pattern baldness is androgen-dependent miniaturisation and loss of frontal and/or vertical hair. It generally starts at the second or third decades and effects almost half of middle-aged men. Therefore, it is quite reasonable that investigators are trying to clarify the presence and extent of a potential interaction between CAD and male pattern baldness.

Although the exact reason for the association between baldness and CAD is unclear, several mechanisms have been proposed. First, elevated androgen levels and increased peripheral sensitivity to androgens have been reported, both in subjects with androgenetic alopecia and CAD [19–21]. Second, it has been suggested that they have common atherosclerosis risk factors such as hypertension, dislipidaemia, smoking, hyperinsulinaemia/insulin resistance, metabolic syndrome and chronic inflammation [22–24]. Third, it has been proposed that the two conditions might share a similar pattern of inheritance [12, 25].

In 1972, Cotton et al. [6] for the first time reported an association between CAD and baldness. They found baldness as a CAD risk factor in 91 patients with overt CAD. However, in 1978, Halim et al. [14] reported that patients with myocardial infarction had no increase in male pattern alopecia. In 1979, Cooke [26] reported that there was no association between CAD and either male pattern alopecia or premature male pattern alopecia in 478 male Caucasian hospital inpatients. In 1993, in a case-control study, Lesko et al. [9] reported that vertex baldness (threefold higher risk of myocardial infarction) but not frontal baldness was associated with myocardial infarction among men younger than 55 years. In the Framingham Heart Study, 30-year follow-up revealed that progression of hair loss during adulthood but not extent of baldness was associated with CAD in men [7]. Additionally, the First National Health and Nutrition Examination Survey reported an association between severe baldness and CAD mortality in men younger than 55 years, but not among older men [8]. In two recent studies, Sharma et al. reported that early-onset androgenetic alopecia in male individuals (less than 40 and 45 years old, respectively) is independently associated with CAD [27, 28]. In contrast, in 2007, Shahar et al. [15] investigated the association of androgenetic alopecia with CAD in 5000 men aged 52–75 years, of whom 767 had a history of myocardial infarction and concluded that male pattern baldness cannot be used as a risk factor for myocardial infarction or asymptomatic atherosclerosis.

To date, there are no other studies in the literature investigating the association between androgenetic alopecia and angiographic CAD severity and collateral development; therefore, we cannot make a direct comparison with published data. However, our results are in agreement with those of Shahar et al. We were not able to detect any relation between presence, severity and age of occurrence of male pattern baldness and Gensini and Rentrop scores, which are important measures of presence and severity of CAD. In our study, although subjects with higher Gensini scores had more frequent and severe baldness, they were older than the group with lower Gensini scores. And after adjusting for age, presence and severity of baldness lost its value in predicting severity of CAD. Additionally, there were no differences in terms of presence and severity of baldness in subjects with and without adequate collateral development. Thus, the previously reported association between CAD and androgenetic alopecia was not confirmed in our angiographic study.

Sasmaz et al. [29] reported that men with vertex-type androgenetic alopecia (*n* = 41) had higher levels of serum lipoprotein (a) and triglyceride when compared with the group with normal hair status (*n* = 36). In their recent study, Dogramaci et al. [30] reported that male individuals with androgenetic alopecia had higher levels of triglycerides and lower levels of high-density lipoprotein. In our study, subjects with vertex baldness but not frontal baldness had significantly higher triglyceride levels supporting the results of the studies by Sasmaz et al. and Dogramaci et al. In our study, the rest of the lipid parameters were comparable between bald and non-bald subjects regardless of the severity of baldness. Interestingly, of the lipid parameters only triglyceride level was higher in subjects with higher Gensini scores. The Physicians’ Health Study, which included 19,112 subjects who were free of CAD at baseline with a follow-up of 11 years, reported that vertex pattern but not frontal baldness was associated with increased risk of coronary events and this association was more robust among men with hypertension or hyperlipidaemia [12]. We did not find such an association in our study. Matilainen et al. [13] reported that men with baldness before the age of 35 years had an increased risk for needing early coronary revascularisation. In our study, presence and severity of androgenetic alopecia before the age of 35 was not associated with angiographic CAD (Gensini and Rentrop scores). Mansouri et al. [31] reported that androgenetic alopecia significantly correlated with CAD and previous history of myocardial infarction in women under the age of 55. We did not study women; however, we did not find an association between androgenetic alopecia and angiographic CAD in men. In a recent meta-analysis, Yamada et al. investigated six observational studies with a total of 36,690 participants. They reported that vertex baldness but not frontal baldness increased the risk of CAD, and the relationship was more robust with severe baldness [32]. As it was a meta-analysis and the setting was completely different, it is not comparable with our results.

Our study has some important points. First, despite the fact that the majority of published data reports an association between androgenetic alopecia and CAD, our study argues against this relationship. Second, our study is the first in the literature to investigate the association between male pattern baldness and angiographic CAD severity and collateral development. Third, our study gathered designs and settings of several previously published studies, such as presence, severity and age of occurrence of male pattern baldness, associated hypertension and hyperlipidaemia. Fourth, regardless of the results, the present study provides more in-depth data in this issue that we believe will shed light for prospective large-scale studies.

Several limitations of our study warrant consideration. First, the sample size of the present study is relatively small, especially for the collateral data. Second, this is a cross-sectional study; therefore, it would provide more valuable information if we were able to follow-up the study population, especially to draw conclusions about clinical coronary events. Third, we asked each participant which category best describes his hair status when he was 35 years old. This might cause recall bias. Fortunately, it was not our primary hypothesis.

## Conclusion

In summary, although the majority of studies reported an association between androgenetic alopecia and CAD, we were not able to detect any relation between presence, severity and age of occurrence of male pattern baldness and Gensini and Rentrop scores, which are important measures of presence and severity of CAD. The potential interaction of male pattern baldness with CAD warrants large-scale prospective studies.
